# Plant Origin, but Not Phylogeny, Drive Species Ecophysiological Response to Projected Climate

**DOI:** 10.3389/fpls.2020.00400

**Published:** 2020-04-07

**Authors:** Zuzana Münzbergová, Veronika Kosová, Renáta Schnáblová, Maan Rokaya, Helena Synková, Daniel Haisel, Nada Wilhelmová, Tomáš Dostálek

**Affiliations:** ^1^Department of Population Ecology, Institute of Botany, Czech Academy of Sciences, Prague, Czechia; ^2^Department of Botany, Faculty of Science, Charles University, Prague, Czechia; ^3^Institute of Experimental Botany, Czech Academy of Sciences, Prague, Czechia

**Keywords:** Balsaminaceae, elevational gradients, genotype × environment interaction, growth chamber experiment, phylogenetic constrains, antioxidants, carotenoids, xanthophyll cycle

## Abstract

Knowledge of the relationship between environmental conditions and species traits is an important prerequisite for understanding determinants of community composition and predicting species response to novel climatic conditions. Despite increasing number of studies on this topic, our knowledge on importance of genetic differentiation, plasticity and their interactions along larger sets of species is still limited especially for traits related to plant ecophysiology. We studied variation in traits related to growth, leaf chemistry, contents of photosynthetic pigments and activity of antioxidative enzymes, stomata morphology and photosynthetic activity across eight *Impatiens* species growing along altitudinal gradients in Himalayas cultivated in three different temperature regimes and explored effects of among species phylogenetic relationships on the results. Original and target climatic conditions determine trait values in our system. The traits are either highly plastic (e.g., APX, CAT, plant size, neoxanthin, β-carotene, chlorophyll *a/b*, DEPSC) or are highly differentiated among populations (stomata density, lutein production). Many traits show strong among population differentiation in degree of plasticity and direction in response to environmental changes. Most traits indicate that the species will profit from the expected warming. This suggests that different processes determine the values of the different traits and separating the importance of genetic differentiation and plasticity is crucial for our ability to predict species response to future climate changes. The results also indicate that evolution of the traits is not phylogenetically constrained but including phylogenetic information into the analysis may improve our understanding of the trait-environment relationships as was apparent from the analysis of SLA.

## Introduction

Knowledge of the relationship between environmental conditions and species traits is an important prerequisite for understanding determinants of community composition and for developing modeling framework allowing to predict species response to novel climatic conditions (Wright et al., 2001; Diaz et al., 2004; Bruelheide et al., 2018). Recently, we have seen an increasing number of studies exploring the trait-environment relationships across global scales (Diaz et al., 2004). To what extent we can see similar patterns at the local scale within sets of closely related species remains to be explored. Exploring the patterns at the finer scales is, however, crucial as the larger scale patterns were shown not to hold at local scales (Wright et al., 2007; Wigley et al., 2016). In addition, Prinzing et al. (2008) demonstrated that trait assembly within communities differs between phylogenetically clustered and phylogenetically diverse communities. This may indicate that also the patterns of trait-environment variation at the within genus level may differ from patterns across range of unrelated species.

Studies on trait-environment relationships are usually working with species traits at the level of species, i.e., assume that the traits are independent of conditions of plant cultivation (trait plasticity) by using traits from large trait databases (Diaz et al., 2004) or combining the genetic and plastic trait components by measuring traits on the local dominant species in the field at each studied site (e.g., Körner and Cochrane, 1985; Hultine and Marshall, 2000; Wright et al., 2001; Hecke et al., 2003; Wigley et al., 2016). Indeed, many studies indicated that values of species traits show large degree of phenotypic plasticity but also different populations may be genetically differentiated from each other and differ in their plastic responses (Weiner, 2004; Poorter et al., 2009; Münzbergová et al., 2017; Münzbergová and Hadincová, 2017). Acknowledging this trait plasticity and also genetic variation in the trait plasticity is crucial for proper understanding of the trait variation patterns across environments (Nicotra et al., 2010).

Majority of studies dealing with trait variation along environmental gradients are focusing on easy to measure traits related to plant morphology and/or simple leaf chemistry (Wright et al., 2001; Diaz et al., 2004; Wigley et al., 2016; Bruelheide et al., 2018). The variation in traits related to plant ecophysiology and the importance of phenotypic plasticity and genetic differentiation in these traits, likely determining species environmental responses, remains to be explored. In addition to exploring patterns of plant morphological traits and simple leaf chemistry, we were thus interested in exploring the contents of photosynthetic and photoprotective pigments, activity of antioxidative enzymes in plant tissue, variation in stomata morphology and photosynthetic efficiency. We were interested in those traits as they represent important parameters describing species stress responses and species adaptation to extreme climatic conditions (e.g., Wildi and Lutz, 1996; Ren et al., 1999; Foyer et al., 2002; Oncel et al., 2004; Singh et al., 2012; Hola et al., 2017; Pavlíková et al., 2017; Szymanska et al., 2017). Production of photosynthetic and photoprotective pigments and their ratios are in direct relation to photosynthetic performance which is crucial for plant metabolism. In adverse conditions, chlorophyll content decreases reflecting damage of the photosynthetic apparatus (Taiz and Zeiger, 2006). On the other hand, xanthophyll pigments and carotenoids are expected to increase as they are protecting chloroplast against negative effects of oxidative stress (Ruban, 2016; Szymanska et al., 2017). In addition, we studied antioxidative enzymes representing defense system that protects whole plant cell. Their activities indicate readiness of plants to cope with adverse conditions (Mittler, 2002, 2006; Szymanska et al., 2017). Variation in stomata morphology informs us on species adjustment to different climatic conditions (Yeh et al., 2012; Yan et al., 2017) and photosynthetic efficiency primarily expresses species stress levels (Benomar et al., 2016).

Many species traits show high levels of phylogenetic conservatism, i.e., their variation is strongly determined by their phylogenetic history (Chomicki et al., 2017; Bawa et al., 2019; Ehmig et al., 2019; Muscarella et al., 2019) and also the species environmental preferences may be strongly phylogenetically constrained (Felsenstein, 1985; Wiens et al., 2010). Understanding the patterns of trait variation across environmental gradients thus requires also understanding of the importance of phylogeny for the trait variation and assessing the effects of the environment on the trait variation after accounting for the phylogenetic patterns. We thus include information on phylogenetic relationships among species into our study. While the importance of phylogeny for trait evolution has been repeatedly studied at the level among plant genera and families (e.g., Moles et al., 2005; Hodgson et al., 2017), similar studies at the intra-generic level are largely missing. We, however, suggest that understanding the importance of phylogeny at the intra-generic level is crucial to understand constrains to evolution during speciation.

To study the trait-environment relationships, we used species of the genus *Impatiens* as the model. Genus *Impatiens* is highly diversified genus of annual or perennial herbs comprising over 1000 species, generally occurring at high altitudes, i.e., more than 1500 m above sea level, distributed mostly in the mountains of the Old-World tropics and subtropics (Yuan et al., 2004; Janssens et al., 2009). One of the biodiversity hotspots of the genus is found in eastern Himalayas and south-east Asia (Song et al., 2003; Yuan et al., 2004; Yu et al., 2016), i.e., the region of our study. By selecting this model system, we aim at understanding the determinants of species performance of a group exposed to highly diverse climatic conditions and at the same time facing strong recent climatic changes.

Specifically, the aim of this study was to assess the effects of climate of plant origin (reflecting genetic differentiation among populations), actual conditions experienced by the plant during its cultivation (reflecting plasticity of the traits, later referred to as target climate) and phylogeny on wide range of species traits. Based on the knowledge previously accumulated in other systems, we predict that both climate of origin as well as target climate will play an important role in determining values of the plant traits in our system and their effects will interact indicating strong variation in plasticity among populations of different origin. As the species from the genus *Impatiens* primarily occur in higher elevations, we specifically expect that plants will show signs of stress when exposed to the warmest temperature and this will be especially true for plants from the highest elevations. As a result, the effects of the interaction between the original and target climate will be stronger than either of their main effects. The expected directions of the responses of the single traits measured, i.e., their values indicating high stress, are in detail explained in the methods and summarized in Table 1. We also predict that stomatal traits will be more affected by plant origin as their values reflect species developmental constrains. In contrast, plant growth, the contents of photosynthetic and photoprotective pigments, antioxidative enzymes and photosynthetic efficiency will show higher effect of current conditions, i.e., plasticity, as these traits are more likely to be modified quickly over the growth of a single individual. Out of these, antioxidative enzymes and photosynthetic efficiency are the most dynamic and their plasticity will thus be the highest (see Table 1). Finally, we predict that more closely related species will possess more similar traits and accounting for phylogenetic relationships among species will thus modify the results on the effects of plant origin. These phylogenetic constrains will be the highest in traits with low plasticity, i.e., in the stomata-related traits.

**TABLE 1:** Summary of predictions and results of the degree of plasticity (effect of target climate), genetic differentiation (effect of climate of origin) and their interaction – genetic differentiation in plasticity.

	Plasticity		Genetic differentiation		Gen. dif. in plast.		Value under stress	Higher stress in
					
	Prediction	Result	Prediction	Result	Prediction	Result	Prediction	Result
Height per week	**↑**	**↑**	↑	0	↑	0	↓	Cold
Leaves per week	**↑**	**↑**	↑	0	↑	0	↓	Cold
Fv/Fm	↓	0	↓	0	↓	0	↓	0
Stomata size	↓	0	↑	0	↓	0	↓	0
Stomata density	↓	0	**↑**	**↑**	↓	0	↑	**Warm**
Neoxanthin	**↑**	**↑**	↓	0	↑	0	↑	Cold
Lutein	↑	0	↓	↑	**↑**	**↑**	↑	Cold
β-carotene	**↑**	**↑**	↓	0	**↑**	**↑**	↓	Cold
Chl.a/b	**↑**	**↑**	↓	0	↑	0	↑	Cold
V + A + Z	↑	0	↓	0	**↑**	**↑**	↑	?
DEPSC	↑	0	↓	↑	**↑**	**↑**	↑	**Warm**
APX	**↑**	**↑**	↓	0	**↑**	**↑**	↑	Cold
CAT	**↑**	**↑**	↓	0	**↑**	**↑**	↑	Cold
SOD isozyme no.	↑	0	↓	0	↑	0	↑	0
SOD activity	↑	0	↓	0	↑	0	↑	0
SLA	↑	n.t.	↑	↓	↑	n.t.	↓	Cold
Nitrogen	↑	n.t.	**↑**	**↑**	↑	n.t.	↓	Cold
Phosphorus	↑	n.t.	**↑**	**↑**	↑	n.t.	↓	Cold

*The directions are highlighted in bold in case the result and prediction go into the same direction. The last two columns represent expected direction of the value under stress, and the conditions under which the expected value was observed (stress in warm conditions shown in bold). 0 indicates non-significant effect (p > 0.05). ? indicates that the stress level depends on plant origin. n.t. indicates not tested for reasons explained in the methods.*

To test these hypotheses, we asked the following questions: (i) What is the effect of climate of plant origin and target climate on the species traits? (ii) Does the importance of plant origin and target climate vary among different traits? (iii) Which conditions are likely stressful for the species based on the different traits? (iv) Do these patterns change after accounting for species phylogenetic relationships?

## Materials and Methods

### Studied System

We sampled 11 species from genus *Impatiens*, Balsaminaceae family, from 30 populations (each population representing one species collected at a single site, Supporting Information 1). All the species are native to Himalayan region (Nepal, India) where their seeds were collected in their natural habitats in the autumn 2016 and 2017. Seeds for each species were collected from at least five mother plants in populations consisting of at least several tens of individuals. Seeds from different maternal plants were kept separately. After collection, the seeds were stored at ambient moisture (approximately 55%) at room temperature (approximately 20°C) for 5 months before the beginning of the experiments. For each population, we recorded year of collection and origin of the population (altitude, longitude, latitude). While we initially collected much larger sample (18 species and 106 populations), many of the seeds showed very low germination and/or extreme seedling mortality, resulting the low final sample size. The determinants of species germination in this system have been in detail explored in our previous study (Veselá et al., 2019) and the issue of low germination and poor seedling survival is also discussed in the discussion. Some of the populations out of the 30 populations used in this study could not be used for all the analyses presented in this paper, so the exact sample size for each trait is described below and shown in detail in Supporting Information 1.

### Experimental Design

Undamaged, fully developed, seeds were exposed to cold wet stratification (5°C) until they started to germinate. After germination, nine germinating seeds from each of five mother plants per population were used to create three identical replicates, each consisting of one pot (5 × 5 × 8.5 cm, filled with a mixture of one part of common garden soil and two parts of sand) with three seedlings. Each replicate was moved to one of three growth chambers (Vötch 1014) differing in their temperature regimes. After 2 weeks, the seedlings were weeded to keep only one seedling per pot. Due to low germination of the collected seeds and low survival of the seedlings in the first few weeks after germination, we had finally only eight species altogether coming from 17 populations (1–4 populations per species) and 187 individuals for all the trait measurements. For all these populations, we were able to cultivate at least three individuals in each growth chamber and they produced enough biomass to be used for all the measurements as described below. For some traits (specific leaf area and nutrient content in the leaves), we were able to use 11 species from 30 populations (1–7 populations per species), as explained below.

The temperature regimes were set to represent the present and future temperatures at localities where *Impatiens* species naturally grow in their native range in Nepal. Temperature regimes were set as follows: (1) cold regime – mean temperature from March to June in 2700 m asl, i.e., in the altitude representing median of higher altitudinal range of *Impatiens* species in Nepal, (2) warm regime – mean temperature from March to June in 1800 m asl, i.e., in the altitude representing median of lower altitudinal range of *Impatiens* species in Nepal, and (3) warm2050 regime – mean temperature from March to June in 1800 m asl as predicted for the year 2050 by global climate model MIRO5C with greenhouse gas concentration trajectory RCP8.5 (Tatebe et al., 2012). Information on the altitudinal range of the *Impatiens* species was obtained from Annotated Checklist of the Flowering Plants of Nepal^1^, which is an updated online version of Press et al. (2000). Temperature data were obtained from WorldClim database (Hijmans et al., 2005).

We used mean temperatures from March to June since this period represents premonsoon period when most *Impatiens* species germinate and start to grow. The course of the temperatures during the day was modeled based on mean, minimum and maximum temperatures which were 12, 6, and 17.5°C for cold regime, 18, 12, and 22.5°C for warm regime and 21, 15, and 25°C for warm2050 regime, respectively (see Supporting Information 2 for details). For all the regimes, the same day length and radiation were used, i.e., 12 h of light (06.00–18.00 h; 250 μmol m^–2^ s^–1^, R/FR = 1.73, PAR/(R + FR) = 8.8, confirmed by direct measurements in the chambers) and 10 h of full dark with a gradual change in light availability in the transition between the light and dark period over 1 h. The light setting was based on the fact that the Himalaya *Impatiens* are growing mainly in mountainous broad-leaved forests and in subalpine coniferous forests thus in conditions with relatively low light intensity (Akiyama et al., 1991). Pots were regularly watered with tap water. We used TOMST loggers (Hemrová et al., 2016; Wild et al., 2019) for detailed monitoring of soil moisture in each chamber and watered the plants when necessary to maintain soil moisture identical across the chambers (corresponding to volumetric moisture of 10%). The growth chambers were also automatically monitoring air humidity maintaining it at the 70% during light and 80% during dark period of the day. All this ensured that our growth chambers likely differ only in temperature and not in any other factors possibly affecting growth of the plants. In the subsequent text, we thus consider that the three growth chambers represent three different target temperatures. This assumption is discussed in the discussion.

### Plant Traits

We measured the following plant traits: plant growth rate, maximum photosystem II (PSII) efficiency (Fv/Fm), stomatal density and stomatal length, content of photosynthetic and photoprotective pigments (chlorophyll *a* and chlorophyll *b*, β-carotene, lutein, neoxanthin, violaxanthin, antheraxanthin and zeaxanthin and calculated their ratios), activity of antioxidative enzymes (L-ascorbate peroxidase, catalase and superoxide dismutase), specific leaf area (SLA) and content of carbon, nitrogen and phosphorus in aboveground plant biomass. All the plant traits (except for the content of carbon, nitrogen and phosphorus in plant biomass and SLA, see below for details) were measured in all the successfully cultivated plants for each population and growth chamber at the time of flower bud production. This allowed us to compare plants in the same developmental stage. For each trait we provide specific prediction of its values assuming that plants exposed to the warmest conditions will suffer the highest stress.

#### Plant Growth

We measured plant height and number of leaves at the time of flower bud production and used this to express plant height and number of leaves produced per week as a measure of plant growth rate. These traits inform us on growth potential of the species. While it would be useful to also obtain data on total flower production of the plants as a measure of fitness, this was not feasible due to very large stature of some of the species, which would not be able to grow in the limited space of the growth chambers and also their very long flowering periods often terminated by autumn frosts in the natural conditions. We expect that stressful conditions will reduce the growth of the plants (Table 1).

#### Maximum Photosystem II Efficiency

Maximum photosystem II (PSII) efficiency (Fv/Fm) was measured using FluorPen FP-100 MAX/USB (Photon System Instruments, Czechia) in dark-acclimated (1 h) plants (Maxwell and Johnson, 2000). The value provides information on overall photosynthetic capacity (Tang et al., 2007; Jahns and Holzwarth, 2012). In healthy leaves, Fv/Fm value is usually close to 0.8. Lower value indicates that a proportion of PSII reaction centers is damaged or inactivated, so called photoinhibition (Ashraf and Harris, 2013). Photoinhibition is commonly observed in plants under stress (Baker and Rosenqvist, 2004; Jahns and Holzwarth, 2012). The plants are thus expected to show reduced Fv/Fm under stressful conditions (Table 1).

#### Stomatal Density and Stomatal Length

Stomatal density and stomatal length were measured using method of Sugano et al. (2010) with modifications to fit our model plants. Leaves were fixed in ethanol:acetic acid = 3:1 for 10 h. Then, the leaves were moved to solution of water:lactic acid:glycerol = 1:1:1 for at least 24 h. Stomatal density was counted as an average of three non-overlapping areas (each 500 × 500 μm). From each of these three areas, three stomata were randomly chosen, and their length was measured, resulting in nine measured stomata per sample. The values were averaged for subsequent analyses. Stomata traits are key drivers of the trade-off between water loss and carbon acquisition by the plant (Raven, 2002; Zhang et al., 2012). The traits are known to be closely linked to temperature (Jumrani et al., 2017), atmospheric CO_2_ concentration (Woodward and Bazzaz, 1988; Woodward et al., 2002), and water availability (Aasamaa and Sober, 2011) but are also related to genome size (Beaulieu et al., 2008) and are determined by genetic background of the plants (Gailing et al., 2008). The effects of all these factors may strongly interact with each other (Chaves et al., 2016; Bertolino et al., 2019). We expect that temperature of plant origin will be the most important driver of stomata size and density and so the plants from the highest altitude will show the lowest density and highest size and the opposite traits in case of originating from the most stressful lowland conditions (see Yan et al., 2017). We also expect these traits will be constrained by species phylogenetic relationships (Table 1).

#### Photosynthetic and Photoprotective Pigments

To measure content of photosynthetic and photoprotective pigments in each plant, we sampled 50 mg of fully developed leaves (3rd leaf from the top). The analyses followed the protocol described in Münzbergová and Haisel (2019). The sampled leaves were frozen in liquid nitrogen, lyophilized for 24 h and stored in a freezer (−80°C) until the analyses. We measured the contents of chlorophyll (chlorophyll *a* and chlorophyll *b*) and carotenoids (β-carotene, lutein, neoxanthin, violaxanthin, antheraxanthin, and zeaxanthin) in all the samples. The contents of all the photosynthetic pigments were analyzed by HPLC (ECOM, Prague, Czech Republic) using a reversed-phase column (Watrex Nucleosil 120-5-C18, 5 μm particle size, 125 × 4 mm, ECOM, Prague, Czech Republic) after extraction of pigments from the leaves with acetone. Elution was carried out for 25 min using a gradient solvent system acetonitrile/methanol/water (80:12:6) followed by methanol:ethylacetate (9:1), the gradient was run at the time of 2–5 min. The flow rate was 1 mL min^–1^ and detection wavelength was 445 nm. Each sample was analyzed twice, and the results were averaged. The contents of all the pigments were expressed as μg/g of dry weight. Chlorophyll *a* and chlorophyll *b* are known to be negatively affected by stress, so their values are expected to decrease with stress (Taiz and Zeiger, 2006). Neoxanthin and lutein have important function in coping with oxidative stress (Dall’Osto et al., 2007; Esteban et al., 2015, Table 1) and their values should thus increase in stressful conditions. We also expect decrease in β-carotene under stress due to its higher consumption in the protection of the photosynthetic apparatus from photooxidation (Munoz and Munne-Bosch, 2018, Table 1).

We also used the pigments to express their composite characteristics. The composite characteristics provide information on changes in different parts of the photosynthetic system. The chlorophyll *a* to chlorophyll *b* ratio (Chl *a*/*b*) is one of basic plant characteristics and provides information about the overall condition of plants and possible stress effects. Chl *a*/*b* tends to increase with increasing stress due to conversion of chlorophyll *b* to chlorophyll *a* (Chen et al., 2017; Table 1). The total content of xanthophyll cycle pigments (V + A + Z, i.e., the sum of violaxanthin, antheraxanthin and zeaxanthin) provides information on plant-protective activity, as these pigments are involved in dynamic interconversion through the operation of the xanthophyll cycle (Forster et al., 2011; Esteban et al., 2015) thus increasing in stressful conditions (Table 1). The de-epoxidation state (DEPS) provides information on xanthophyll cycle activity and the degree of violaxanthin to zeaxanthin conversion. It was defined as (0.5A + Z)/(V + A + Z) following (Pospisilova et al., 2000). DEPSC is the value of DEPS relative to total chlorophyll production. Higher DEPSC value indicates higher levels of stress response (DemmigAdams and Adams, 1996; Fernandez-Marin et al., 2011; Szymanska et al., 2017; Table 1).

#### Antioxidative Enzymes

Soluble enzymes were extracted from 0.5 g of frozen fully developed leaves homogenized with mortar and pestle in 2.5 mL buffer (0.1 M Tris-HCl, 1 mM dithiothreitol, 1 mM Na_2_EDTA, 1% Triton X-100, 5 mM ascorbic acid, pH 7.8) and a small amount of PVP (polyvinylpyrrolidone). Samples were incubated on ice in the dark for 30 min and centrifuged (16,000 g, 10 min, 4°C). Pellets were discarded, and supernatants were divided into Eppendorf tubes, frozen in liquid nitrogen and stored at −70°C (Lubovska et al., 2014) until used to analyze total soluble protein content according to method described in Bradford (1976) and enzyme activities (L-ascorbate peroxidase, catalase, and superoxide dismutase) as described below.

L-ascorbate peroxidase (APX, EC 1.11.1.11) and catalase (CAT, EC 1.11.1.6) belong to enzymes detoxifying H_2_O_2_. Activity of those enzymes is an important indicator of antioxidative status of plants in changing environment. L-ascorbate peroxidase and catalase activities were assayed spectrophotometrically in protein extracts in stirred cells maintained at 25°C (spectrophotometer U-3300, Hitachi, Japan). L-ascorbate peroxidase activity was determined by monitoring a decrease of ascorbate concentration as absorbance change at 290 nm (A_290_) according to Nakano and Asada (1981). One unit of L-ascorbate peroxidase activity was expressed as Δ A_290_ per mg^–1^ (total soluble protein) min^–1^. Catalase activity was detected as a decrease of hydrogen peroxide concentration monitored as absorbance change at 240 nm (A_240_; Chance and Maehly, 1955). One unit of catalase activity was defined as Δ A_240_ per mg^–1^ (total soluble protein) min^–1^. The measurements of activity were repeated four times and the resulting values were averaged for further analyses.

Superoxide dismutase (SOD, EC 1.15.1.1.) isozyme patterns and activities were obtained after separation by 12% non-denaturing polyacrylamide gel electrophoresis (PAGE). Aliquots of crude extracts from leaf tissue samples corresponding to 35 μg of protein per lane were used for analysis. SOD isozymes were detected *in situ* in the gel by photochemical nitroblue tetrazolium (NBT) staining method according to Beaucham and Fridovic (1971). The different isozymes of SOD were distinguished according to sensitivity to inhibition by 2 mM KCN and 5 mM H_2_O_2_. Mn-SOD is resistant to KCN and H_2_O_2_, Fe-SOD is resistant to KCN but inhibited by H_2_O_2_, and both inhibitors inhibit Cu-Zn-SOD (Fridovich, 1986). Stained gels were scanned and densitograms were created and analyzed in *ImageJ* program (Wayen Rasband, U.S. National Institutes of Health, Bethesda, MD, United States^2^). The relative activity of total SOD was estimated as the sum of intensities of individual bands expressed as peak areas. Number of isozymes was counted and assigned according to their type after the inhibition by KCN or H_2_O_2_.

L-ascorbate peroxidase, superoxide dismutases and catalase represent the most important enzymes eliminating harmful reactive oxygen species (ROS). Accumulation of ROS is an important consequence of unfavorable conditions (Van Breusegem and Dat, 2006; Farrant and Ruelland, 2015; Lopez-Orenes et al., 2017; Soares et al., 2019). Proper function of the defense system is thus crucial for efficient functioning of plant cells (Das and Roychoudhury, 2014; Kapoor et al., 2015). Information on activity of these enzymes provides information on stress tolerance of the plants (Sairam and Saxena, 2000; Singh et al., 2012). Plants growing in more stressful conditions are thus expected to show higher activities of these enzymes (Table 1).

#### Specific Leaf Area and Nutrient Content in the Leaves

Due to high amount of biomass required for analyses of nutrient content in the leaves (0.5 g dry weight), we had to merge the leaves across samples and analyze mixed samples for each population. For this reason, we could not test the effect of growth chamber on these variables. The chemical analyses were performed in the Analytical laboratory of the Institute of Botany, Academy of Sciences of the Czech Republic. The contents of nitrogen and carbon were analyzed following (Ehrenberger and Gorbach, 1973). The content of phosphorus was analyzed spectrophotometrically at a wavelength of 630 nm (Unicam UV4-100, Cambridge, United Kingdom; Olsen et al., 1954) after digestion in HNO_3_ and H_2_O_2_. While preparing material for these analyses, we measured SLA of these leaves and used the information on SLA as another response variable. Because we combined the material across growth chambers for this analysis, we could include more species and populations. Specifically, we included 12 species from 31 populations (Supporting Information 1). We expect that plants in the most stressful conditions will show the lowest nutrient accumulation and the lowest SLA (Table 1).

### Data Analyses

In total, our dataset contained 22 dependent variables measured within each population and growth chamber. Before any analyses, the variables not fitting the condition of normality and homoscedasticity were transformed as necessary. This included log transformation of the content of L-ascorbate peroxidase and catalase and square root transformation of plant height and number of leaves per week and stomatal density. Using these data, we calculated pair-wise correlation among all the dependent variables (Supporting Information 3). Due to high correlations (*r* ≥ 0.7), some of the variables, namely chlorophyll *a*, chlorophyll *b*, chlorophyll *a* + *b*, antheraxanthin, violaxanthin, zeaxanthin, and DEPS have been excluded from further analyses. When excluding variables, we preferred to retain the composite variables over the simple variables. We thus used 15 different depended variables for further analyses in total. These include V + A + Z, DEPSC, plant height per week, number of leaves per week, Chl *a*/*b*, β-carotene, lutein, neoxanthin, L-ascorbate peroxidase, catalase, stomata length and density, SOD isozyme number, and SOD activity and Fv/Fm.

In addition, we had data on content of nitrogen, phosphorus and C/N ratio in the leaves and their SLA. These variables were measured for each population but summed across growth chambers (see above). Using these data, we calculated pair-wise correlation among all the dependent variables (Supporting Information 4). Due to high correlations (*r* ≥ 0.7), the C/N ratio has been excluded from further analyses.

To test the effects of climate of origin, we categorized our populations into three altitudinal ranges. The first group included populations growing below 2300 m asl (5 populations, further referred to as low). The second included populations occurring between 2301 and 2680 m asl (8 populations, further referred to as intermediate). The third included populations growing above 2680 m asl (5 populations, further referred to as high). We classified the altitude into categories as the altitudinal gradient was not evenly covered by the populations. However, testing altitude as a continuous variable provided qualitatively similar results. Then we tested the effect of climate of origin and target climate (climate of the growth chamber) on the single species traits measured in each growth chamber using mixed effect model with population as a random factor. All the dependent variables were assumed to follow Gaussian distribution (some after transformation as stated above), except for number of SOD isozymes following Poisson distribution. In addition to analyzing the whole dataset, we also performed pair-wise comparison among all the categories using the same models. Results of these tests were used to visualize differences among the categories within the graphs.

In addition to using the single traits, we also performed a principle component analysis (PCA) as implemented in Canoco 5.0 (Šmilauer and Lepš, 2014) using all the initially measured plant traits (22 in total) and used position of each sample on the first two ordination axes as composite sample characteristics. All the traits have been standardized (mean = 0, *SD* = 1) before entering this analysis. The first PCA axis distinguished plants with high chlorophyll production and low DEPS and DEPSC values from plants with the opposite character combinations and explained 34% of the variation in the trait values. The second axis distinguished plants with high V + A + Z values and stomatal length from plants with the opposite trait values and explained 10.6% of the variation in the trait values (Supporting Information 5). We used these two PCA axes as a composite dependent variable in the tests described above. As the second axis has never shown any significant response to any of the predictors, it is not presented further.

We used analyses of variance to test the effect of altitude on content of nitrogen, phosphorus and SLA. We did not have to use mixed effect models with population as random factor here as we had only one value for each population in this case and did not have data for the different growth chambers. In this dataset, there were 12, 10, and 9 populations belonging to the low, intermediate and high altitudinal belt, respectively.

To assess the effect of phylogenetic relationships among the species on the patterns observed, we used data on ITS phylogeny of the plant group developed for the purpose of another study (Líblová et al. in prep.). Phylogenetic distance matrix describing the relationships among populations was decomposed into its eigenvectors using PCoA as suggested by Diniz et al. (1998) and Desdevises et al. (2003) using the R-package ***“***ape***”*** (Paradis et al., 2004). The first two eigenvectors explained 76% of the variability in the data. They were included as co-variables in all the above analyses in order to correct for phylogenetic autocorrelation and to compare the effects of phylogeny to the effects of original and target climate. In addition, we also explored the effects of interaction between phylogeny and original and target climate on all our response variables.

The effects of climate of origin, target climate and their interaction have been tested on 15 independent traits measured on the same experimental plants. Theoretically, we should apply the Bonferroni correction and reduce the conventional p-level from 0.05 to 0.003 (Dunn, 1961). We decided to use a modification of this approach, the sequential Bonferroni correction (Holm–Bonferroni correction, Rice, 1989) as it is considered as less conservative. Still any such correction is considered as too conservative (e.g., Moran, 2003; Garcia, 2004; Gotelli and Ellison, 2004) and many studies have thus not applied any correction (e.g., Münzbergová, 2007; Bowman et al., 2008; Scheepens and Stocklin, 2013). We believe that strict adherence to the correction would also go against an important aim of our study, i.e., assess the importance of species target and origin across wide range of traits and describe the different drivers in the different traits. We thus report and illustrate results both with and without this correction (see Husáková and Münzbergová, 2016; Münzbergová et al., 2017 for similar approach). We do the same for the variables compared only among different altitudes of plant origin.

### Methodological Considerations

It may be argued that our experiment is pseudoreplicated as the growth chambers may theoretically differ in a range of other variables (e.g., light intensity) leading to possible spurious treatment effects (Hurlbert, 1984). However, as we in detail explain in our previous study (Münzbergová et al., 2017), this argument is not valid in our case as in many other studies using experiments in growth chambers or multiple common gardens. Our analyses may thus be considered as valid. For extended discussion of this issue (see Supporting Information 6).

## Results

**FIGURE 1:**
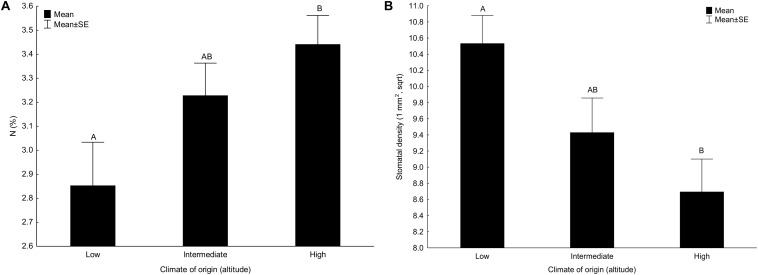
Effect of climate of origin expressed as altitudinal category on **(A)** content of nitrogen (N) and **(B)** stomatal density. Columns sharing the same letter are not significantly different from each other (*p* > 0.05).

Content of lutein, nitrogen (Figure 1A) and phosphorus (Tables 2, 3) significantly increased with altitude (representing climate of origin) while density of stomata (Figure 1B) decreased with altitude (Table 2). All the other main effects of altitude were not significant (Tables 2, 3).

**TABLE 2:** Results of mixed effect models testing the effect of climate of origin (characterized by altitudinal category), target climate (i.e., conditions in the growth chamber) and their interaction on the single traits.

		PCA1	Height.per.week	Leaves.per.week	Stomata density	Neoxanthin	Lutein	β-carotene	Chl.a/b	V + A + Z	DEPSC	APX	CAT
Origin	*F*	0.61	1.75	1.27	**5.92**	2.01	**4.11**	1.84	0.01	1.01	0.81	0.91	0.65
	*p*	0.442	0.199	0.271	**0.017**	0.172	**0.054**	0.183	0.905	0.325	0.373	0.349	0.429
	*R*^2^	–	–	–	**0.35**	–	**0.14**	–	–	–	–	–	–
Target	*F*	**3.93**	**4.35**	**16.96**	0.77	**14.93**	1.12	**14.74**	**9.85**	0.79	**12.72**	**16.3**	**7.97**
	*P*	**0.049**	**0.039**	< 0.001*	0.382	< 0.001*	0.291	< 0.001*	0.002*	0.375	< 0.001*	< 0.001*	0.005*
	*R*^2^	**0.36**	**0.43**	**0.74**	–	**0.79**	–	**0.37**	**0.31**	–	**0.22**	**0.47**	**0.63**
Origin x	*F*	**4.65**	2.25	1.37	1.76	1.89	**23.09**	**8.62**	1.07	**4.28**	**10.18**	**5.94**	**4.08**
target	*P*	**0.033**	0.136	0.244	0.186	0.171	< 0.001*	0.004*	0.302	**0.04**	0.002*	**0.016**	**0.045**
	*R*^2^	**0.43**	–	–	–	–	**0.81**	**0.63**	–	**0.31**	**0.65**	**0.17**	**0.32**

Results for stomata length, SOD isozyme number, SOD activity and Fv/Fm are not shown in the table as none of the predictors was significant (p > 0.05). Year of sampling was used as a covariate and population as a random factor in all the models. PCA1 represents first ordination axis from a multivariate analysis of the traits. R^2^ indicates deviance explained out of the total deviance explained by the model and is provided only for significant effects. Significant values (p ≤ 0.05) are in bold. Values marked with * are significant even after sequential Bonferroni correction.

**TABLE 3:** Results of analysis of variance exploring the effects of (A) climate of origin expressed as altitudinal category, and (B) phylogeny (phylo) and climate of origin on content of nitrogen (N), phosphorus (P) and specific leaf area (SLA).

	Nitrogen			Phosphorus			SLA		
			
	*F*	*p*	*R*^2^	*F*	*p*	*R*^2^	*F*	*p*	*R*^2^
(A) **Origin**	**7.59**	0.010*	**0.21**	**15.65**	<0.001*	**0.35**	3.38	0.076	0.10
(B) Phylo	0.38	0.545	–	0.01	0.925	–	0.06	0.813	–
**Origin**	**6.54**	0.017*	**0.19**	**14.38**	0.001*	**0.35**	**4.27**	0.049*	**0.14**

R^2^ indicates variance explained out of the total variance of the data. Significant values (p ≤ 0.05) are in bold. Values marked with * are significant even after sequential Bonferroni correction.

**FIGURE 2:**
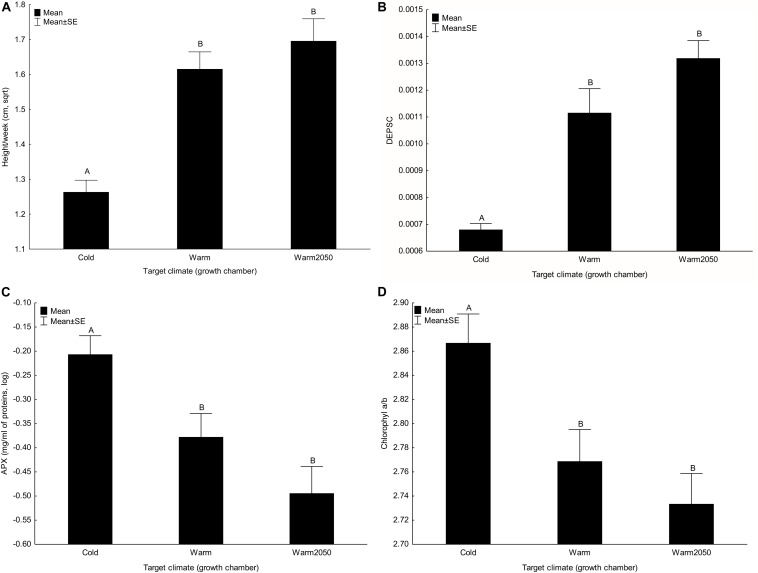
Effect of target climate (growth chamber) on **(A)** height increase per week, **(B)** DEPSC, **(C)** L-ascorbate peroxidase and **(D)** chlorophyll *a/b*. Columns sharing the same letter are not significantly different from each other (*p* > 0.05).

Plant height (Figure 2A) and number of leaves per week and DEPSC (Figure 2B) significantly increased with target temperature. In contrast, content of neoxanthin, β-carotene, activities of L-ascorbate peroxidase (Figure 2C) and catalase and the chlorophyll *a/b* ratio (Figure 2D) decreased with increasing target temperature. These effects were also apparent from significant result for the composite trait. All the other main effects of target temperature were not significant (Table 2).

**FIGURE 3:**
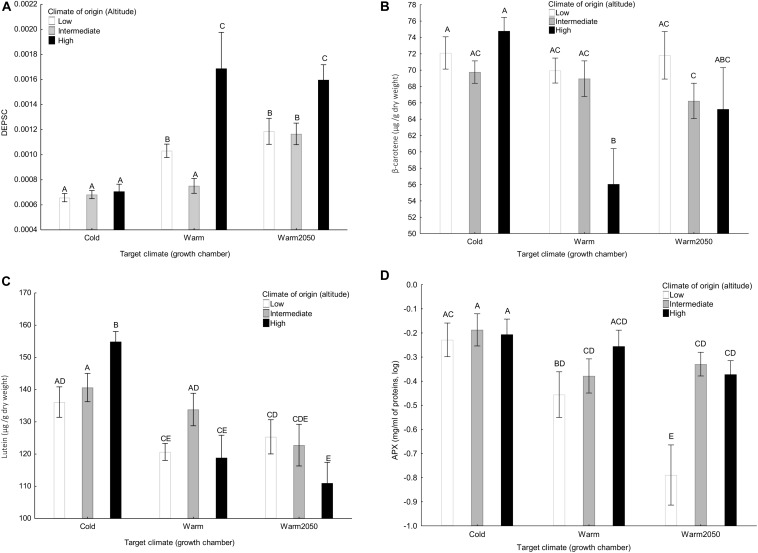
The effect of climate of origin expressed as altitudinal category and target climate (growth chamber) on **(A)** DEPSC, **(B)** β-carotene, **(C)** lutein, and **(D)** L-ascorbate peroxidase separately in the three target growth chambers. Columns sharing the same letter are not significantly different from each other (*p* > 0.05).

All the variables except for plant growth (plant height and number of leaves per week), neoxanthin content, stomatal length and density, SOD isozyme number and SOD activity, Fv/Fm and chlorophyll *a/b* showed significant interaction between climate of origin and target climate (Table 2). V + A + Z increased with altitude in the cold chamber but did not show any relationship with altitude in the other chambers. DEPSC increased with altitude in the warm and warm2050 chamber but did not depend on altitude in the cold chamber (Figure 3A). Content of β-carotene (Figure 3B) did not show any relationship with altitude in the cold chamber and decreased with altitude in the two warmer chambers. Content of lutein (Figure 3C) increased with altitude in the cold chamber and decreased in the warm2050 chamber with no relationship in the warm chamber. Content of L-ascorbate peroxidase (Figure 3D) and catalase showed a significant positive relationship with altitude in the warm and warm2050 chambers but not in the cold chamber.

**FIGURE 4:**
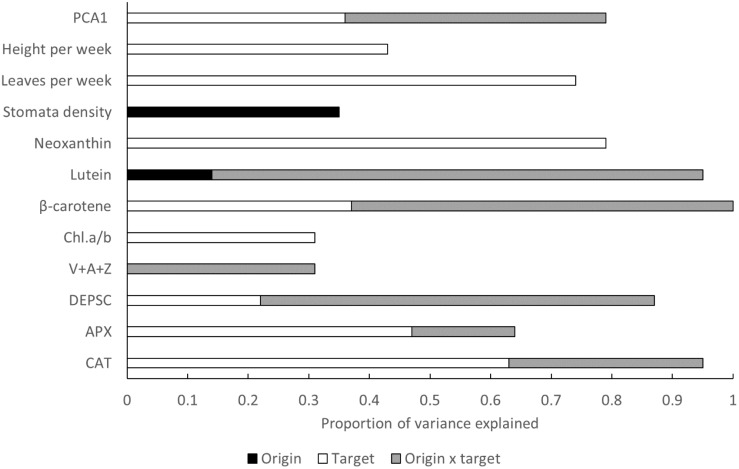
Proportion of variance explained by plant origin expressed as altitudinal category, target (growth chamber) and origin × target out of the variance explained by the whole model. PCA1 represents the composite trait summarizing all the measured response variables.

Among trait comparison indicates that the highest proportion of variance explained is related to phenotypic plasticity (effect of target environment, Figure 4). In several traits, the highest proportion of variance can be attributed to origin × target interaction indicating genetic differentiation in phenotypic plasticity (Figure 4). The effects of origin are overall very rare (Figure 4).

### Effects of Phylogeny

Phylogeny had significant effect on plant growth expressed as number of leaves per week (*F* = 21.03, *p* = 0.001), Chl *a*/*b* (*F* = 5.89, *p* = 0.033), but not on any other variables (not shown for variables in Table 2, for N, P, and SLA provided in Table 3). The effect of phylogeny never interacted with the effects of climate of origin (*p* > 0.05 in all cases). In two cases, we found significant interaction between phylogeny and target climate. This was true for fluorescence (*F* = 6.91, *p* = 0.009) and content of carotenoids (*F* = 23.35, *p* < 0.001).

Including phylogeny into the models only slightly modified the variance explained by the models but did not change the significance patterns. The only exception was the result for SLA. In this case, phylogeny did not have any significant effect, but its inclusion led to significant positive effect of altitude of plant origin (Table 3).

## Discussion

The results indicate that both original and target climate determine trait values in our system, but interestingly, we never detected significant main effects of both target and origin for a specific trait. This indicates that the traits are either highly plastic (such as L-ascorbate peroxidase, catalase, plant growth, neoxanthin, β-carotene, chlorophyll *a/b*, and DEPSC) or are highly differentiated among populations from different environments (stomatal density and lutein production). In addition, many of the traits show strong interactions between target and origin indicating high among population differentiation in the degree of plasticity and direction in the response to the environmental changes. Out of these traits, lutein, DEPSC, and β-carotene showed the highest variance explained by this interaction (Figure 4). All this suggests that different processes determine the values of the different traits and separating the importance of genetic differentiation and plasticity is crucial for our ability to predict species response to future climate changes. The results also indicate that the evolution of the traits is not phylogenetically constrained but including phylogenetic information into the analysis may improve our understanding of the trait-environment relationships as was apparent from the analysis of SLA in our system. Some of the traits did not show any significant response to any predictor. These results are discussed only in Supporting Information 7.

### Effect of Climate of Origin

Concentration of nitrogen and phosphorus significantly increased with altitude of plant origin representing a proxy for climate of origin. This is in line with the prediction that alpine plants are adapted to maintain higher concentration of nutrients in their leaves compared to lowland species (Körner and Cochrane, 1985; Friend et al., 1989; Körner, 1999; Oleksyn et al., 2002, but see Hultine and Marshall, 2000; Richardson, 2004). This may be explained by the need to accumulate resources quickly in the usually harsh environments with short growing season (Körner, 1999). Reduced nutrient acquisition in the plants from lower altitudes may be also a response to higher herbivore pressure in lowlands (Rasmann et al., 2014; Dostálek et al., 2016, 2018; Rokaya et al., 2016). Higher concentrations of nitrogen and phosphorus and reduced C/N in the leaves is known to increase leaf herbivory (Münzbergová and Skuhrovec, 2013), and their reduced concentration may thus constitute part of the anti-herbivore syndrome of the lowland plants (Knappová et al., 2018; Kergunteuil et al., 2019). Importantly, our results indicate that these patterns can be detected also at the level of among species within a single genus and they are independent of phylogenetic relationships among the species. By exploring these patterns on material grown in the common conditions, this result also indicates that the patterns have a genetic basis and are thus not purely due to phenotypic plasticity. This is important as previous studies looking at such a relationship in the field found mixed results (e.g., Hultine and Marshall, 2000; Richardson, 2004). Most previous studies were not able to separate the genetic and plastic component of the variation due to collecting data directly in the field. Genetic differentiation in the ability to acquire more nutrients is thus an important mechanism of species adaptation to higher altitudes, together with species plastic response to temperature changes pointing to the same direction (Weih and Karlsson, 2001).

After accounting for the phylogenetic relationships among species, we also detected significant positive effect of altitude on SLA. The pattern contrasts to the results of Körner and Cochrane (1985) and Hultine and Marshall (2000) indicating decreasing SLA with altitude. As SLA was in our case positively correlated with nitrogen content in the leaves, our results also contrast with statement that nitrogen content is inversely correlated with SLA (Körner and Cochrane, 1985). Similar pattern, i.e., increasing SLA in higher altitudes was also observed by Anderson and Gezon (2015). They suggest that shorter growing seasons at higher altitudes may favor traits that enable rapid development including high SLA. This may possibly also explain the pattern observed in our system.

We also detected significant decrease in stomatal density with increasing altitude of plant origin. This is in line with our expectation that the plants originating from warmer conditions will produce more stomata due to necessity of effective transpirational cooling and with range of studies demonstrating positive effect of increasing temperature to stomatal density reviewed in Yan et al. (2017). On the other hand, our results contrast with studies demonstrating increase in stomatal density with increasing altitude (also reviewed in Yan et al., 2017). Nevertheless, altitudinal gradients largely vary in environmental factors such as CO_2_ concentration, temperature, light intensity, and soil water availability, all of which may also strongly affect stomatal development (Woodward et al., 2002; Casson et al., 2009; Aasamaa and Sober, 2011). The interactions among these factors with altitude may thus cause the inconsistencies observed. Note, however, that our sites along the elevational gradient show differences in temperature, but do not show any directional changes in precipitation and solar radiation during the growing season (Supporting Information 1).

The only other dependent variable significantly responding to the main effect of altitude was production of lutein increasing with increasing altitude of plant origin. This is in line with the results of Hecke et al. (2003). They, however, found significant effects of altitude also on neoxanthin, β-carotene, DEPS a V + A + Z (similarly e.g., Oncel et al., 2004; Acuna-Rodriguez et al., 2017). The significant effect for lutein but not other pigments may be linked to the fact that lutein is the most abundant xanthophyll in higher plants (Jahns and Holzwarth, 2012) and has thus the highest potential to show detectable variation among populations.

The lack of significant relationships with altitude for majority of response variables in our study is likely due to interaction between altitude and growth chamber masking the possible main effects of altitude. In addition, our temperature treatments did not simulate the effect of high irradiance that is an additional stressful factor for plants in higher altitudes (Oncel et al., 2004, but such a pattern was not detected in our dataset, Supporting Information 1). Note that the interaction between altitude and growth chamber could not be tested in foliar N and P and SLA as these values were integrated across the different growth chambers. As majority of the studies showing main effects of altitude on the various traits were field studies exploring the relationships using material directly occurring in the field (e.g., Körner and Cochrane, 1985; Hultine and Marshall, 2000; Hecke et al., 2003), their results in fact integrated the possible plastic and genetic responses and did not allow to detect such interactions (but see Zhang et al., 2012).

### Effect of Target Climate

In line with our expectation and previous studies (e.g., Pigliucci, 2001; Nicotra et al., 2010; Raabova et al., 2011; Campbell et al., 2019), we detected many significant effects of target climate indicating high phenotypic plasticity in majority of the traits. This indicates that the plasticity is more important than genetic differentiation for most of the traits measured in this study. High phenotypic plasticity is important for species response to novel conditions as it allows populations to buffer detrimental effects of environmental change allowing enough time for evolutionary changes to occur (e.g., Jump and Penuelas, 2005; Anderson et al., 2012; Padilla et al., 2013). Interestingly, the only traits that showed significant main effect of altitude of origin, namely stomatal density and lutein production, did not show any significant effect of target climate, indicating no overall plasticity in these traits. In contrast, none of the traits showing significant target effects showed at the same time significant effects of origin. This contrasts to a range of previous studies indicating that both genetic differentiation and plasticity contribute to explaining species trait variation (e.g., Franks et al., 2014; Münzbergová et al., 2017).

Both L-ascorbate peroxidase and catalase showed lower activities in higher temperature. As the production of both of these enzymes indicates species response to oxidative stress (Willekens et al., 1995, 1997), it seems to suggest that plants are more stressed when grown in colder conditions (Hasanuzzaman et al., 2013). As these responses are primarily driven by plants from low altitude, these patterns are discussed below together with the interactions between conditions of origin and target climate.

Target climate also significantly contributed to explaining variation in plant growth (height and number of leaves per week). While this relationship simply reflects that plants grow more rapidly under warmer conditions (e.g., Chapin and Shaver, 1996; Rustad et al., 2001; Münzbergová et al., 2017), it is surprising that target climate was the only significant predictor in this case. This contrasts with previous studies indicating that plant growth is also strongly determined by conditions of plant origin (Münzbergová et al., 2017), but corresponds, e.g., to conclusions of Ran et al. (2013) and Datta et al. (2017). The absence of the origin pattern in this study may be because these traits strongly vary among species. Even though these traits do not show any phylogenetic constrains, the among species differentiation may override the effects of altitude of origin.

Target climate has also been the only significant predictor of chlorophyll *a/b* ratio. Lower chlorophyll *a/b* ratio with increasing temperature was previously observed also by Saez et al. (2019). They suggest that this modification implies an increase in the antenna/core PSII proportion and could contribute to management of the light energy before it reaches the reaction center, maintaining the photochemical activity of the plant unaffected (see also Ensminger et al., 2006). The changes in the ratio may also reflect drought stress as under drought stress the reduction of chlorophyll *b* is greater than that of chlorophyll *a* increasing the chlorophyll *a/b* ratio (Jaleel et al., 2009; Ashraf and Harris, 2013). In the *Impatiens* species, i.e., species adapted to high alpine conditions and experiencing no water stress in the experiment, we suggest that the changes in the chlorophyll *a/b* ratio are more likely linked to the cold adaptation.

Finally, also the content of neoxanthin was only affected by target temperature. While the content of neoxanthin has been previously shown to vary with conditions of plant cultivation (e.g., Martins et al., 2016), we are not aware of any study that would assess the effects of plant origin on its value.

Target climate in our study is simulated in three growth chambers. As we describe in the methods, we attempted to ensure that the growth chambers only differ in the temperature and thus interpret the differences as the effects of temperature. Such an approach has two types of problems. First, as we have only one chamber per treatment, the target temperatures represent pseudoreplication and the main effects of target climate should be interpreted keeping this in mind (for more details see methods and Supporting Information 6). Second, changes in temperature never happen in isolation in natural conditions and are accompanied by changes in other factors, e.g., air humidity. By ignoring this fact, the conclusions on the effects of temperature may differ from those estimated under natural conditions. It thus should be kept in mind that despite we describe the effects of target temperature in the paper, the main value of our findings lies in the knowledge of the effects of climate of origin and the interactions between original and target climates.

### Interactions Among Original and Target Climate

Significant interactions between original and target climate indicate significant differentiation in trait plasticity suggesting that the populations have high potential to cope with changing conditions (Franks et al., 2014). Our results indicate such significant interactions for DEPSC, V + A + Z, β-carotene, lutein, catalase, L-ascorbate peroxidase and the composite trait. For DEPSC, lutein, and β-carotene this interaction explained more variation than either of the two main effects. The importance of interactions between target and origin for the production of photoprotective pigments are in line with Ramirez-Valiente et al. (2015) suggesting the importance of climate of origin in shaping species response abilities. The high variance explained by this interaction in this system contrasts to previous studies (e.g., Gugger et al., 2015; Münzbergová et al., 2017) suggesting that the main effects of target and origin tend to be more important. This indicates high potential of the *Impatiens* species to adapt to novel environmental conditions.

The activity of L-ascorbate peroxidase and catalase seems to strongly respond to target climate in plants coming from low altitudes, while high-altitude plants produce constitutively high values of the enzymes. This seems to suggest that the high-altitude plants are ready to cope with stress at any occasion, while the low-altitude plants only induce such response in colder conditions. This is in line with the fact that plants growing in optimal conditions are not challenged so their antioxidant activities are lower than in plants growing in stressful environments (Bowler and Fluhr, 2000; Foyer et al., 2002; Scandalios, 2005; Singh et al., 2012; Hola et al., 2017). This thus suggests that the alpine conditions are stressful from the point of view of the plants at least based on L-ascorbate peroxidase and catalase production. Higher activation of antioxidant system in plants coming from higher altitude is also reported previously (e.g., Wildi and Lutz, 1996; Ren et al., 1999; Oncel et al., 2004; Wang et al., 2009). High altitude represents stress for plants not only due to low temperature, but also due to high light intensity that may lead to an inactivation of D1 protein of the PSII. It can cause an imbalance between light absorption and light use, leading to the accumulation of reactive oxygen species (Wang et al., 2009), that have to be scavenged by an efficient antioxidative system. However, different species may activate different components of the antioxidative enzymes in case of stress (Streb et al., 1997).

In contrast to L-ascorbate peroxidase and catalase, the value of DEPSC strongly increases with target temperature in plants from high altitude, while the increase is much smaller in plants from lower altitude. As higher DEPSC value indicates higher levels of stress response (DemmigAdams and Adams, 1996; Fernandez-Marin et al., 2011; Szymanska et al., 2017), the results suggest that plants are more stressed under higher temperature and this effect is much higher in high-altitude plants.

The value of V + A + Z was independent of altitude of origin in plants growing in warm growth chamber. In the cold chamber, the content of V + A + Z increased with altitude of plant origin. In contrast, in the warm2050 chamber, the content of V + A + Z decreased with altitude of plant origin. Due to their photoprotective function, higher values are expected to indicate higher stress levels (Forster et al., 2011; Esteban et al., 2015). It thus seems that low-altitude plants were the most stressed in the warmest conditions while high-altitude plants were most stressed in the coldest conditions. While this seems counterintuitive, it may be because protection via V + A + Z is relatively costly and may be a strategy used only in plant response to stress they often experience in their home sites.

### Effects of Phylogeny

In contrast to our expectations, we found only very few weak effects of phylogeny on the species traits. We also found two significant interactions with target climate indicating that plants of different origin show differential response to conditions of the growth chambers. We, however, did not find any such an interaction with climate of plant origin. All this suggests that both current and original environmental conditions are key determinants of the trait values and that the phylogenetic constrains do not really limit trait evolution within the genus.

The absence of clear phylogenetic patterns in our dataset may have multiple explanations. Previous studies indicating strong phylogenetic conservatism in species traits were usually done on much larger sets of species using traits extracted from the databases (e.g., Ackerly and Nyffeler, 2004; Moles et al., 2005; Kerkhoff et al., 2006; Ackerly, 2009). Increasing our sample size may thus possibly increase the likelihood of detecting phylogenetic patterns. In addition, most of the previous studies providing evidence for phylogenetic conservatism in species traits worked with larger sets of species across many genera and families (e.g., Moles et al., 2005; Hodgson et al., 2017) suggesting that the phylogenetic constrains are caused by similar developmental and design constraints in related species (Ackerly and Donoghue, 1995; Figueroa and Armesto, 2001). Absence of such a pattern at the intra-generic study may thus suggest that evolution within the genus is dynamic enough not to limit species potential to adapt to novel conditions. Finally, the absence of phylogenetic signal in our study may also be because the traits we studied are easier to modify than traits showing high phylogenetic conservatism in previous studies. For example, many previous studies showed strong phylogenetic conservatism in seed size (e.g., Zhang et al., 2004; Moles et al., 2005; Barak et al., 2018) and seed size was in fact shown to be highly phylogenetically conserved in another study in the same system of the genus *Impatiens* (Veselá et al., 2019). Further studies on phylogenetic patterns of eco-physiological traits within a single genus are thus needed to assess the generality of our findings. The fact that including phylogenetic information into our study led to finding a significant and unexpected effect of altitude on SLA also suggest that including phylogenetic information into future studies on species traits is indeed important.

### Limitations of the Study

The results of this study deal exclusively with performance of grown-up plants, but completely ignore the seed germination and seedling establishment, i.e., the most sensitive parts of the life cycle (e.g., Knapp et al., 2008; Walck et al., 2011; Dreesen et al., 2014). As indicated in the methods, our initial sampling has been much more extensive and majority of our material did not germinate and/or survive the initial stages of plant life. The drivers of germination of this seed material have been explored in our previous study (Veselá et al., 2019). Among others, Veselá et al. (2019) demonstrated that seeds experiencing warmer temperature, especially leading to shortening of the stratification period, exhibit lower seed germination. This suggests that warming can have negative effects on this part of the life-cycle. Some species, however, did not germinate at all and could not be analyzed in Veselá et al. (2019). The lack of their germination could be caused for example by their suboptimal storage during their transport from Nepal or bad timing of their collection in the field. We assume that bad timing of seed collection is especially likely, as the seeds of all the species disperse by ballistic dispersal based on explosion of the fruits (Chapman and Gray, 2012). As a result, it is extremely difficult to collect the seeds at the very narrow window of time when they are already mature, but have not yet dispersed.

In addition, some of the seeds germinated but died soon after germination. Low seedling survival could be caused, e.g., by presence of some pathogens in our experimental facilities, which do not occur in the native sites. It is thus possible that the high mortality is an artifact of the experimental conditions and does not match the patterns, which would be observed in the field. For both seed germination and seedling survival, it is also possible that our plants have suffered reduced fitness due to low genetic diversity of the populations or due to poor seed development due to unfavorable conditions at the maternal sites. Both these mechanisms have been previously suggested to affect seed germination and seedling survival (e.g., Dostálek et al., 2010; Münzbergová and Plačková, 2010; Veselá et al., 2020). We, however, assume that the other mechanisms suggested above are more likely as they concern many species and populations. In any case, the determinants of survival of the species require further exploration. The results of this study thus need to be interpreted with caution as the early stages of plant life cycle may be the key determinant of the response of *Impatiens* species to changing climates.

### Implications for Species Response to Future Climate

The plants grow the best under the warmest conditions. At the same time, plants exposed to the warmest conditions show the lowest production of L-ascorbate peroxidase and catalase, indicating the lowest levels of oxidative stress in plants in the warmest conditions. These traits may thus suggest that the *Impatiens* species are likely to profit from the ongoing climate warming. This may be linked to the fact that the group is of tropical origin (Shajitha et al., 2016). In contrast, the parameters based on production of photosynthetic and photoprotective pigments indicate increased stress in the warmest conditions. This can be seen from increasing DEPSC and decreasing neoxanthin and β-carotene with the target temperature (DemmigAdams and Adams, 1996; Havaux and Niyogi, 1999; Niyogi, 1999; Fernandez-Marin et al., 2011; Szymanska et al., 2017; Munoz and Munne-Bosch, 2018). As the changes in the pigments went to the opposite direction than the changes in plant growth, it may suggest that the changes in the pigment production of the plants under different conditions is an efficient strategy to protect the plants. The observed decrease in chlorophyll *a/b* may indicate increased stress under colder conditions or maximalization of light use efficiency under warmer conditions, in both cases indicating that warmer conditions are more favorable for the plants.

The above may indicate that the *Impatiens* species from the Himalayas will profit from the ongoing climate changes. This may, however, not be the full story for several reasons. Firstly, in our previous study, Veselá et al. (2019), demonstrated that plants exposed to warmer temperature, especially to the shortening of the stratification period, exhibit lower seed germination. Secondly, Dostálek et al. (2019), demonstrated, that the same plants become more palatable when exposed to the warmer temperature and may thus suffer higher levels of herbivory. Thirdly, our conclusions are based on the fact that faster growth indicates higher fitness, and this may not be true. However, our observations suggest that close relationship between plant growth and fitness is likely as the plants grow and flower continuously until the first frosts. This was also the reason, why we were unable to properly estimate fitness of the plants. Finally, increased temperature is also likely to increase biomass production of the whole plant communities (Van De Velde et al., 2019) and the annual species of *Impatiens* may lose suitable habitats for germination and establishment and be outcompeted by longer lived perennial species. All this indicates that species response to novel climatic conditions require studying wide range of species traits including the early stages of plant-life cycle to get the full picture of possible species responses.

## Data Availability Statement

The datasets generated for this study are available on request to the corresponding author.

## Author Contributions

ZM, TD, and MR conceived the idea and designed the experiment. MR collected the field material. TD, VK, RS, HS, DH, and NW collected the data and wrote methodology of the respective parts. ZM analyzed the data and wrote the manuscript. TD provided extensive comments during manuscript preparation. All co-authors commented on the manuscript and approved the final version.

## Conflict of Interest

The authors declare that the research was conducted in the absence of any commercial or financial relationships that could be construed as a potential conflict of interest.

## Footnotes

1 http://www.efloras.org/flora_page.aspx?flora_id=110

2 http://rsb.info.nih.gov/ij
