# Supplementary material for: Plant Origin, but Not Phylogeny, Drive Species Ecophysiological Response to Projected Climate
Source: Front Plant Sci. 2020 Apr 7;11:400. doi: 10.3389/fpls.2020.00400 (PMC7154175; doi:10.3389/fpls.2020.00400)
Supplement: Supplementary file 1 [file Data_Sheet_1.docx]

Supporting Information

Supporting Information 1. List of species and populations used in the study; altitude (m a.s.l.), latitude (N) and longitude (E) and temperature, precipitation and sol. Number 1 in column Main indicates that the given population was used in the main analyses (most traits across the 3 growth chambers). Number 1 in column Chemistry indicates that the given population was used for studying SLA and leaf chemistry.

Supporting Information 2. Course of temperatures within each growth chamber over 24 hours. For all the regimes, the same day length and radiation were used, i.e. 12 h of 60% light (06.00–18.00 h; 250 μmol m-2 s-1) and 10 h of full dark with a gradual change in light availability in the transition between the light and dark period over 1 h.

Supporting information 3. Pairwise correlation matrix among all the dependent variables measured in each growth chamber. Variables shown in grey have been excluded from further analyses due to their high correlations (r ≥ 0.7) with the other variables.

Supporting information 4. Pairwise correlation matrix among all the dependent variables measured on material collected across the growth chambers. Variables shown in grey have been excluded from further analyses due to their high correlations (r ≥ 0.7) with the other variables.

Supporting information 5. Result of principle component analysis (PCA) as implemented in Canoco 5.0 (Šmilauer and Lepš 2014) using all the plant traits as dependent variables. All the traits have been standardized before entering the analysis. The first (horizontal) PCA axis explained 34% of the variation in the trait values, the second (vertical) axis explained 10.6% of the variation in the trait values.

Supporting Information 6. Detailed consideration of the possible pseudoreplication issue in the experiment. The text largely corresponds to our text published previously as Supporting Information of a paper Münzbergová et al. (2017).

Supporting Information 7. Discussion of results of traits that did not show any significant response to any predictor.

Supporting Information 1. List of *Impatiens* species and populations used in the study; altitude (m a.s.l.), latitude (N) and longitude (E) and climatic conditions. Number 1 in column Main indicates that the given population was used in the main analyses (most traits across the 3 growth chambers). Number 1 in column Chemistry indicates that the given population was used for studying SLA and leaf chemistry. Data on mean, minimum and maximum temperatures, mean solar radiation and total precipitation for studied localities in the premonsoon period (March to June) were obtained from WorldClim database (Fick et al. 2017).

* Sensu (Pusalkar and Singh 2010)

** Sensu (Akiyama and Ohba 2016)

*** Sensu (Akiyama and Ohba 2016). Before revision by (Akiyama and Ohba 2016) usually called *I. scabrida.*

**References**

Fick, S. E., Hijmans, R.J. (2017) WorldClim 2: New 1-Km Spatial Resolution Climate Surfaces for Global Land Areas". International Journal of Climatology 37: 4302–4315.

Supporting Information 2. Course of temperatures within each growth chamber over 24 hours. For all the regimes, the same day length and radiation were used, i.e. 12 h of 60% light (06.00–18.00 h; 250 μmol m-2 s-1) and 10 h of full dark with a gradual change in light availability in the transition between the light and dark period over 1 h.


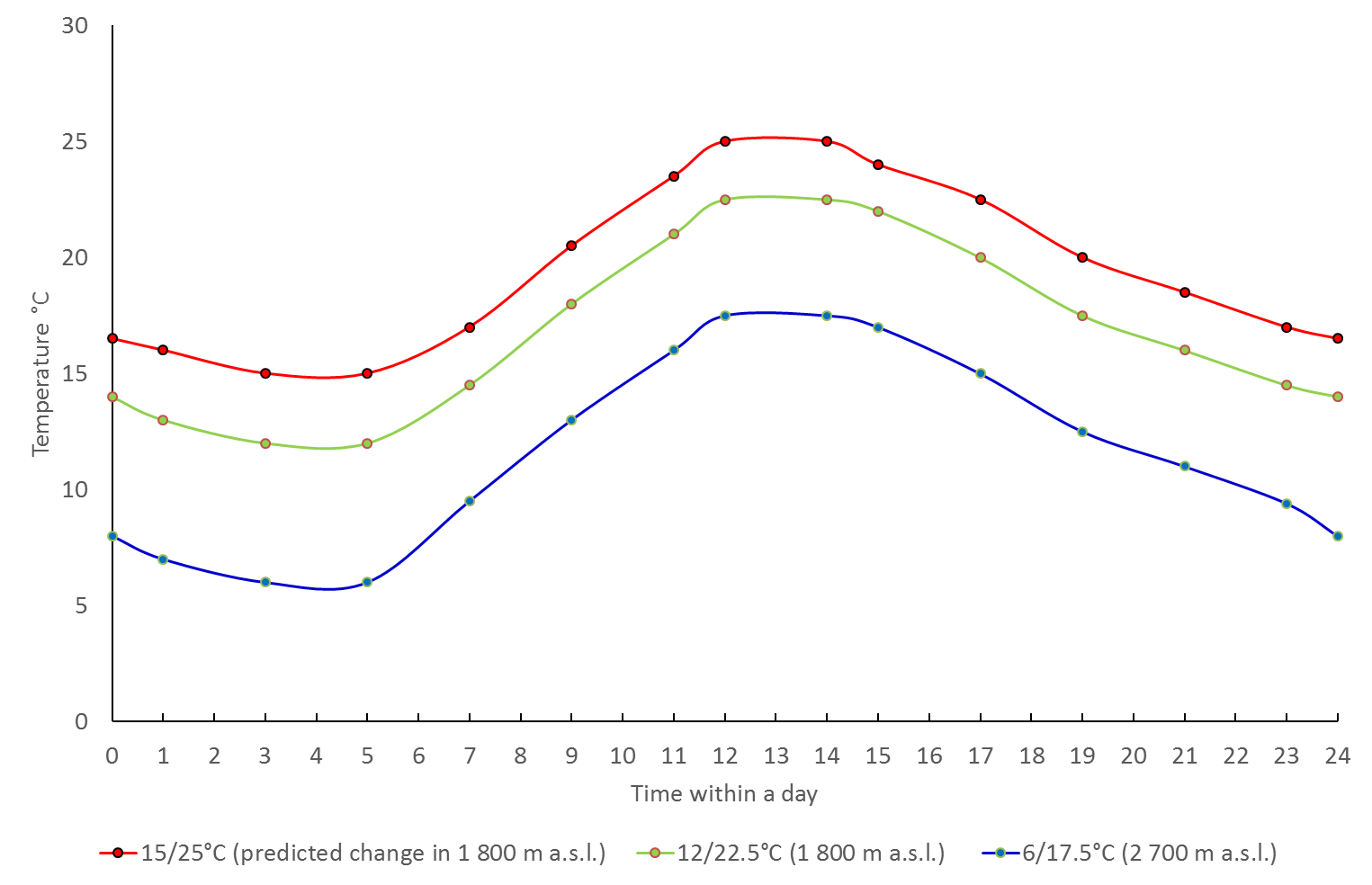


Supporting information 3. Pairwise correlation matrix among all the dependent variables measured in each growth chamber. Variables shown in grey have been excluded from further analyses due to their high correlations (r ≥ 0.7) with the other variables.

|  | Antheraxanthin | β-carotene | DEPS | DEPSC | Fluorescence | Chl.a | Chl.a/b | Chl.a+b | Chl.b | Lutein | APX | CAT | Neoxanthin | Height.per.week | Leaves.per.week | Stomata density | Stomata length | Violaxanthin | V.A.Z | Zeaxanthin | SOD number | SOD quantity |
| --- | --- | --- | --- | --- | --- | --- | --- | --- | --- | --- | --- | --- | --- | --- | --- | --- | --- | --- | --- | --- | --- | --- |
| Antheraxanthin | - | -0.18 | 0.75 | 0.56 | 0.11 | -0.52 | -0.54 | -0.49 | -0.40 | -0.31 | -0.07 | 0.06 | -0.31 | 0.18 | 0.26 | 0.09 | 0.13 | -0.38 | 0.21 | 0.77 | -0.12 | -0.14 |
| β-carotene | -0.18 | - | -0.26 | -0.58 | 0.04 | 0.56 | 0.19 | 0.57 | 0.61 | 0.32 | 0.08 | 0.01 | 0.31 | -0.05 | 0.01 | 0.03 | -0.11 | 0.19 | 0.04 | -0.31 | -0.09 | 0.00 |
| DEPS | 0.75 | -0.26 | - | 0.79 | -0.03 | -0.73 | -0.48 | -0.71 | -0.67 | -0.28 | -0.04 | 0.02 | -0.45 | 0.28 | 0.05 | 0.20 | -0.13 | -0.81 | -0.32 | 0.84 | -0.14 | -0.03 |
| DEPSC | 0.56 | -0.58 | 0.79 | - | -0.04 | -0.89 | -0.55 | -0.89 | -0.87 | -0.45 | -0.02 | 0.04 | -0.52 | 0.18 | 0.07 | 0.12 | 0.02 | -0.57 | -0.14 | 0.78 | -0.03 | -0.09 |
| Fluorescence | 0.11 | 0.04 | -0.03 | -0.04 | - | 0.06 | 0.10 | 0.05 | 0.02 | 0.07 | -0.15 | 0.00 | 0.09 | -0.12 | 0.04 | 0.08 | 0.11 | 0.15 | 0.23 | 0.07 | -0.09 | -0.03 |
| Chl.a | -0.52 | 0.56 | -0.73 | -0.89 | 0.06 | - | 0.62 | 0.99 | 0.94 | 0.47 | 0.12 | -0.01 | 0.46 | -0.16 | -0.14 | -0.20 | 0.07 | 0.58 | 0.21 | -0.70 | -0.04 | 0.05 |
| Chl.a/b | -0.54 | 0.19 | -0.48 | -0.55 | 0.10 | 0.62 | - | 0.57 | 0.35 | 0.57 | -0.07 | -0.27 | 0.24 | -0.27 | -0.39 | -0.04 | -0.11 | 0.23 | -0.15 | -0.58 | -0.04 | 0.06 |
| Chl.a+b | -0.49 | 0.57 | -0.71 | -0.89 | 0.05 | 0.99 | 0.57 | - | 0.97 | 0.46 | 0.13 | 0.01 | 0.47 | -0.16 | -0.10 | -0.20 | 0.05 | 0.58 | 0.20 | -0.68 | -0.04 | 0.07 |
| Chl.b | -0.40 | 0.61 | -0.67 | -0.87 | 0.02 | 0.94 | 0.35 | 0.97 | - | 0.37 | 0.15 | 0.08 | 0.47 | -0.10 | 0.00 | -0.21 | 0.07 | 0.57 | 0.25 | -0.61 | -0.03 | 0.07 |
| Lutein | -0.31 | 0.32 | -0.28 | -0.45 | 0.07 | 0.47 | 0.57 | 0.46 | 0.37 | - | 0.02 | -0.17 | 0.25 | -0.32 | -0.44 | -0.15 | -0.04 | 0.19 | -0.04 | -0.34 | -0.18 | -0.01 |
| APX | -0.07 | 0.08 | -0.04 | -0.02 | -0.15 | 0.12 | -0.07 | 0.13 | 0.15 | 0.02 | - | 0.55 | -0.01 | 0.11 | -0.27 | -0.28 | 0.08 | 0.08 | 0.07 | 0.01 | 0.02 | 0.11 |
| CAT | 0.06 | 0.01 | 0.02 | 0.04 | 0.00 | -0.01 | -0.27 | 0.01 | 0.08 | -0.17 | 0.55 | - | 0.05 | 0.06 | -0.02 | -0.15 | 0.07 | 0.06 | 0.12 | 0.09 | 0.12 | 0.01 |
| Neoxanthin | -0.31 | 0.31 | -0.45 | -0.52 | 0.09 | 0.46 | 0.24 | 0.47 | 0.47 | 0.25 | -0.01 | 0.05 | - | -0.11 | -0.15 | -0.01 | -0.19 | 0.31 | 0.08 | -0.42 | -0.16 | 0.07 |
| Height.per.week | 0.18 | -0.05 | 0.28 | 0.18 | -0.12 | -0.16 | -0.27 | -0.16 | -0.10 | -0.32 | 0.11 | 0.06 | -0.11 | - | 0.38 | 0.05 | -0.01 | -0.28 | -0.18 | 0.15 | -0.11 | 0.00 |
| Leaves.per.week | 0.26 | 0.01 | 0.05 | 0.07 | 0.04 | -0.14 | -0.39 | -0.10 | 0.00 | -0.44 | -0.27 | -0.02 | -0.15 | 0.38 | - | 0.15 | 0.16 | 0.04 | 0.14 | 0.05 | 0.00 | -0.16 |
| Stomata density | 0.09 | 0.03 | 0.20 | 0.12 | 0.08 | -0.20 | -0.04 | -0.20 | -0.21 | -0.15 | -0.28 | -0.15 | -0.01 | 0.05 | 0.15 | - | -0.09 | -0.28 | -0.24 | 0.08 | 0.02 | 0.04 |
| Stomata length | 0.13 | -0.11 | -0.13 | 0.02 | 0.11 | 0.07 | -0.11 | 0.05 | 0.07 | -0.04 | 0.08 | 0.07 | -0.19 | -0.01 | 0.16 | -0.09 | - | 0.25 | 0.34 | 0.00 | 0.08 | -0.18 |
| Violaxanthin | -0.38 | 0.19 | -0.81 | -0.57 | 0.15 | 0.58 | 0.23 | 0.58 | 0.57 | 0.19 | 0.08 | 0.06 | 0.31 | -0.28 | 0.04 | -0.28 | 0.25 | - | 0.80 | -0.41 | 0.07 | 0.01 |
| V.A.Z | 0.21 | 0.04 | -0.32 | -0.14 | 0.23 | 0.21 | -0.15 | 0.20 | 0.25 | -0.04 | 0.07 | 0.12 | 0.08 | -0.18 | 0.14 | -0.24 | 0.34 | 0.80 | - | 0.17 | -0.01 | -0.07 |
| Zeaxanthin | 0.77 | -0.31 | 0.84 | 0.78 | 0.07 | -0.70 | -0.58 | -0.68 | -0.61 | -0.34 | 0.01 | 0.09 | -0.42 | 0.15 | 0.05 | 0.08 | 0.00 | -0.41 | 0.17 | - | -0.15 | -0.03 |
| SOD number | -0.12 | -0.09 | -0.14 | -0.03 | -0.09 | -0.04 | -0.04 | -0.04 | -0.03 | -0.18 | 0.02 | 0.12 | -0.16 | -0.11 | 0.00 | 0.02 | 0.08 | 0.07 | -0.01 | -0.15 | - | 0.22 |
| SOD quantity | -0.14 | 0.00 | -0.03 | -0.09 | -0.03 | 0.05 | 0.06 | 0.07 | 0.07 | -0.01 | 0.11 | 0.01 | 0.07 | 0.00 | -0.16 | 0.04 | -0.18 | 0.01 | -0.07 | -0.03 | 0.22 | - |

Supporting information 4. Pairwise correlation matrix among all the dependent variables measured on material collected across the growth chambers. Variables shown in grey have been excluded from further analyses due to their high correlations (r ≥ 0.7) with the other variables.

|  | N | P | C/N | SLA |
| --- | --- | --- | --- | --- |
| N | - | 0.52 | **-0.92** | 0.40 |
| P | 0.52 | - | -0.48 | 0.39 |
| C/N | **-0.92** | -0.48 | - | -0.52 |
| SLA | 0.40 | 0.39 | -0.52 | - |

Supporting information 5. Result of principle component analysis (PCA) as implemented in Canoco 5.0 (Šmilauer and Lepš 2014) using all the plant traits as dependent variables. All the traits have been standardized before entering the analysis. The first (horizontal) PCA axis explained 34% of the variation in the trait values, the second (vertical) axis explained 10.6% of the variation in the trait values.


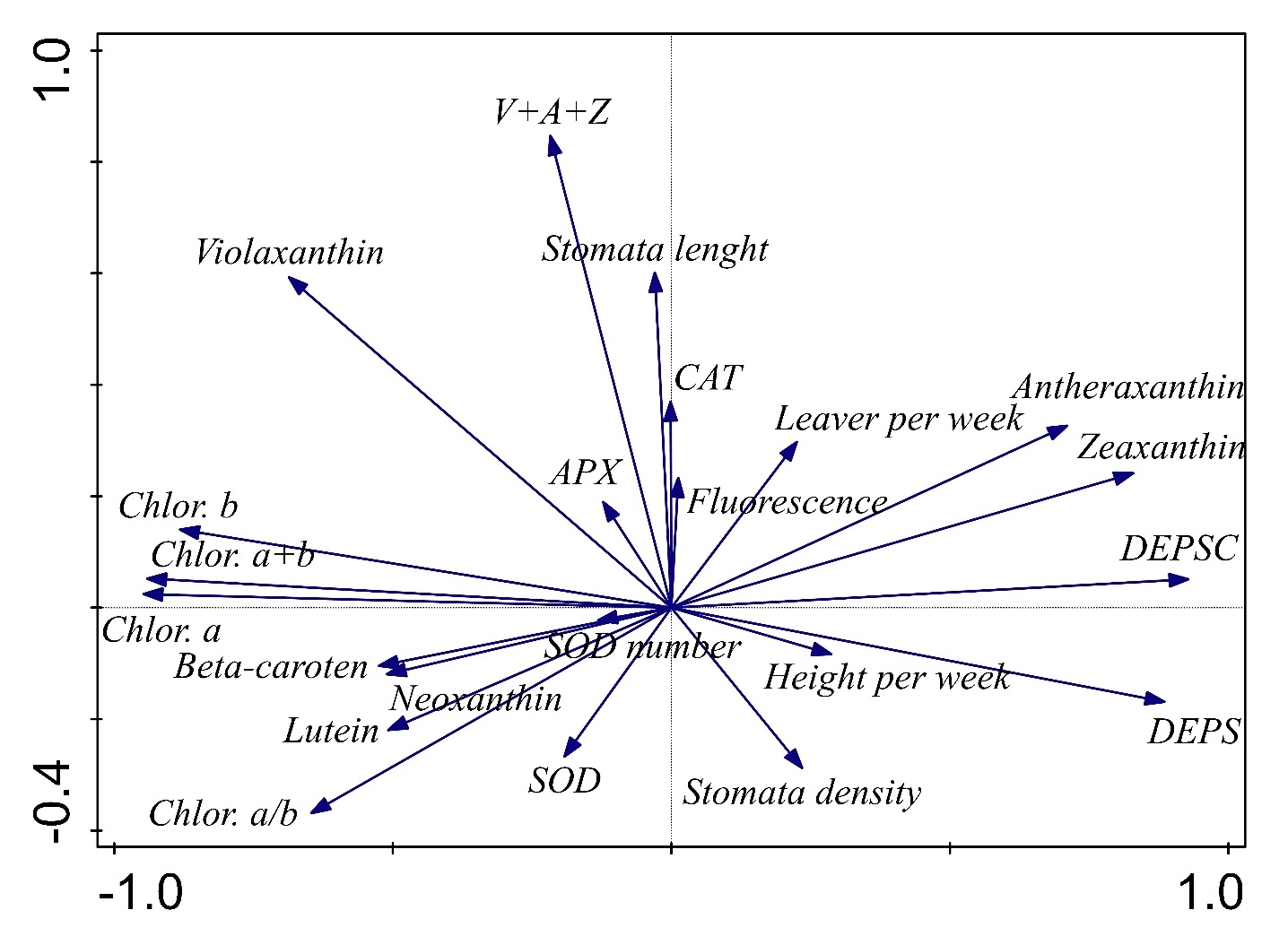


Supporting Information 6. Detailed consideration of the possible pseudoreplication issue in the experiment. The text largely corresponds to our text published previously as Supporting Information of a paper Münzbergová et al. (2017).

Our experiment is an isolative segregation type of pseudoreplication as defined by Hurlbert, (1984) as the growth chambers may theoretically differ in a range of other variables as well (e.g. light intensity) leading to possible spurious treatment effects (Hurlbert, 1984). As a defence against similar criticism, some authors (e.g., Bezemer et al., 1998; VuorinenNerg and Holopainen, 2004; VuorinenNergIbrahim et al., 2004; Souther et al., 2012) regularly transferred plants among the growth chambers and reset their conditions. This, however, does not solve the issue as the theoretical differences between the growth chambers could interact with actual conditions in the growth chambers which are gradually changing, so it would even not fully eliminate the effect of growth chamber. In addition, as the plants are moved simultaneously among the growth chambers, such an experiment could still be considered pseudoreplicated (Johnson et al., 2016). Moreover, resetting the growth chambers would disrupt the plant daily cycles and the course of temperature and humidity which needs some time to stabilize. Also the physical transfer of plants through the building may cause a wide range of other unwanted negative effects. In addition, measurements of light intensity and climatic conditions within the growth chambers supported the producer statement that the growth chambers are largely identical in their function with larger differences between the edges and centres of single growth chambers than between growth chambers. As a result, randomizing the plants within the growth chambers, as we did on a monthly basis, was much more important than transferring plants between the growth chambers. Thus, we can be quite confident that the different temperature and moisture regimes applied in the different growth chambers are the main differences between our treatments (see also Davies and Gray, 2015 for similar argumentation).

The conclusions of Hurlbert, (1984) on pseudoreplication in growth chamber experiments have been criticised by a range of authors (e.g., Oksanen, 2001, Johnson et al., 2016). Later, also Hurlbert, (2004) concluded that such experiments can be analysed with standard statistical approaches as long as the interaction term is used as estimate of the error term to test the main effect. In our experiment, the effect manipulated at the growth chamber level, i.e. the target environment, is not the effect of primary interest. Rather, we were primarily interested in the effect of original environment, which is well replicated and the interaction between the original and target environment. In such a case, using the standard error terms is well justified. Thus in line with a range of other studies using similar setting either with unreplicated gardens in different elevations (Scheepens and Stocklin, 2013; Gugger et al., 2015) or growth chambers (Bezemer et al., 1998; Cavieres and Arroyo, 2000; Souther et al., 2012; Matias and Jump, 2014; Zhang et al., 2014), we suggest that such studies are useful by allowing to separate genetic differentiation of plants from their phenotypic plasticity and to study the effect of specific climatic variables without confounding effects of other naturally varying factors.

While using multiple growth chambers per treatment would indeed be the best approach, it is unrealistic due to very high costs of buying as well as maintaining each chamber. Based on all the arguments above, we can be confident that our approach is powerful to explore the importance of original environment and the interaction between target and origin and thus to understand the responses of the target species to changing climates.

References

Bezemer, T. M., Thompson, L. J. & Jones, T. H. (1998) *Poa annua* shows inter-generational differences in response to elevated CO2. *Global Change Biology,* **4,** 687-691.

Cavieres, L. A. & Arroyo, M. T. K. (2000) Seed germination response to cold stratification period and thermal regime in *Phacelia secunda* (Hydrophyllaceae) - Altitudinal variation in the mediterranean Andes of central Chile. *Plant Ecology,* **149,** 1-8.

Davies, G. M. & Gray, A. (2015) Don't let spurious accusations of pseudoreplication limit our ability to learn from natural experiments (and other messy kinds of ecological monitoring). *Ecology and Evolution,* **5,** 5295-5304.

Gugger, S., Kesselring, H., Stoecklin, J. & Hamann, E. (2015) Lower plasticity exhibited by high- versus mid-elevation species in their phenological responses to manipulated temperature and drought. *Annals of Botany,* **116,** 953-962.

Hurlbert, S. H. (1984) Pseudoreplication and the design of ecological field experiments. *Ecological Monographs,* **54,** 187-211.

Hurlbert, S. H. (2004) On misinterpretations of pseudoreplication and related matters: a reply to Oksanen. *Oikos,* **104,** 591-597.

Johnson, S. N., Gherlenda, A. N., Frew, A. & Ryalls, J. M. W. (2016) The importance of testing multiple environmental factors in legume-insect research: replication, reviewers, and rebuttal. *Frontiers in Plant Science,* **7**.

Matias, L. & Jump, A. S. (2014) Impacts of predicted climate change on recruitment at the geographical limits of Scots pine. *Journal of Experimental Botany,* **65,** 299-310.

Münzbergová Z, Hadincová V, Skálová H, Vandvik V (2017) Genetic differentiation and plasticity interact along temperature and precipitation gradients to determine plant performance under climate change. *Journal of Ecology,* **105**, 1358-1373. doi: 10.1111/1365-2745.12762

Oksanen, L. (2001) Logic of experiments in ecology: is pseudoreplication a pseudoissue? *Oikos,* **94,** 27-38.

Scheepens, J. F. & Stocklin, J. (2013) Flowering phenology and reproductive fitness along a mountain slope: maladaptive responses to transplantation to a warmer climate in *Campanula thyrsoides*. *Oecologia,* **171,** 679-691.

Souther, S., Lechowicz, M. J. & McGraw, J. B. (2012) Experimental test for adaptive differentiation of ginseng populations reveals complex response to temperature. *Annals of Botany,* **110,** 829-837.

Vuorinen, T., Nerg, A. M. & Holopainen, J. K. (2004) Ozone exposure triggers the emission of herbivore-induced plant volatiles, but does not disturb tritrophic signalling. *Environmental Pollution,* **131,** 305-311.

Vuorinen, T., Nerg, A. M., Ibrahim, M. A., Reddy, G. V. P. & Holopainen, J. K. (2004) Emission of *Plutella xylostella*-induced compounds from cabbages grown at elevated CO2 and orientation behavior of the natural enemies. *Plant Physiology,* **135,** 1984-1992.

Zhang, H. X., Yu, Q., Huang, Y. X., Zheng, W., Tian, Y., Song, Y. T., Li, G. D. & Zhou, D. W. (2014) Germination Shifts of C-3 and C-4 Species under Simulated Global Warming Scenario. *Plos One,* **9**.

Supporting Information 7. Discussion of results of traits that did not show any significant response to any predictor.

Despite the many significant effects of target and original climate and their interactions on various species traits, we also found a range of traits showing no significant patterns. One of those is chlorophyll fluorescence expressed as Fv/Fm. Overall the values of Fv/Fm were only slightly below 0.8 (indicating healthy leaves with no stress due to photoinhibition) going down to only 0.61 indicating only limited photoinhibition ((Ashraf and Harris 2013)). Fv/Fm was also the variable with the lowest CV among all the variables tested (0.036). While (Saez et al. 2019) found significant variation of Fv/Fm value with target temperature, they also suggested that the fluctuations are in fact very low with most of the values being close to 0.8. High stability of the values may thus suggest that none of the cultivating conditions in fact led to an important stress in our species at least when judged from the photosynthetic activity.

Another trait not showing any significant response was stomatal length. In our dataset, stomatal length was the variable with the third lowest CV (0.12) indicating its high stability. In a previous study, (Zhang et al. 2012b) found only significant effect of origin in one species and no significant effect on stomatal length in another species. Absence of effect of altitude on stomatal length, despite significant effects on stomatal density and negative correlation between the two traits was also observed by (Anderson and Gezon 2015). In their study, stomatal length, however, turned to be vary plastic as it significantly changed with target climate. The degree of genetic differentiation and plasticity in stomatal length is thus highly system specific.

Also, SODs activity and isozyme composition did not show any significant variation. We found more or less steady activity and about five different isozymes which seems to be typical for *Impatiens* species ((Lall et al. 1999) (Milosevic et al. 2012)). Although some researchers found only Mn-SOD and Cu/Zn-SODs isozymes in the *Impatiens* species ((Lall and Nikolova 2002)), we could also distinguish Fe-SOD isozyme in most samples similarly as (Milosevic et al. 2015) in *Impatiens walleriana*. SOD plays a central role in defense against reactive oxygen species (ROS) converting superoxide radicals to H_2_O_2._  As ROS are common byproducts of plant metabolism, SOD steady activity helps to keep a production and scavenging ROS in a balance under optimal conditions. Under conditions of environmental abiotic or biotic stress, overall SOD activity increases, and new isozymes can be formed. Previous studies demonstrated that drought, CO_2_ enrichment, high light, and biotic stress can affect SODs in *Impatiens* species ((Antonic et al. 2016) (Zhang et al. 2012a) (Lall and Nikolova 2002) (Milosevic et al. 2012)); the increase of total SOD activity ranged between 11–40% and some new isozymes were also found. In our experiment, the temperature stress has been apparently too low to induce any SOD responses. The absence of any effect of plant origin seems to be in line with the fact that SOD production is highly plastic showing strong effects of target climate and low genetic differentiation among populations (Fusari et al. 1997). In another system, it has been shown that activity of SOD increases in roots but not in leaves of plants from higher altitudes (Ren et al. 1999). It is thus also possible that we could find differences in case of using other parts of the plants for the analysis.

References

Anderson JT, Gezon ZJ (2015) Plasticity in functional traits in the context of climate change: a case study of the subalpine forb Boechera stricta (Brassicaceae). Global Change Biology 21:1689-1703. doi: 10.1111/gcb.12770

Antonic D et al. (2016) Effects of exogenous salicylic acid on *Impatiens walleriana* L. grown in vitro under polyethylene glycol-imposed drought. South African Journal of Botany 105:226-233. doi: 10.1016/j.sajb.2016.04.002

Ashraf M, Harris PJC (2013) Photosynthesis under stressful environments: An overview. Photosynthetica 51:163-190. doi: 10.1007/s11099-013-0021-6

Fusari A et al. (1997) Non-optimal growth temperatures and antioxidants in the leaves of Sorghum bicolor (L.) Moench. I. Long term acclimation. Phyton-Annales Rei Botanicae 37:71-79

Lall N, Nikolova RV (2002) Developmental changes of superoxide dismutase, peroxidase and catalase isozyme profiles in leaves of *Impatiens flanaganiae*  Hemsl. associated with variations in light intensity. South African Journal of Botany 68:518-524

Lall N, Nikolova RV, Bosa AJN (1999) Changes in activities of superoxide dismutase peroxidase and catalase from leaves of *Impatiens flanaganiae* in response to light intensity. South African Journal of Botany 65:255-259. doi: 10.1016/s0254-6299(15)30992-3

Milosevic S, Lojic M, Antonic D, Cingel A, Subotic A (2015) Changes of antioxidative enzymes in *Impatiens walleriana*  L. shoots in response to genetic transformation. Genetika-Belgrade 47:71-84. doi: 10.2298/gensr1501071m

Milosevic S et al. (2012) Response of antioxidative enzymes to long-term Tomato spotted wilt virus infection and virus elimination by meristem-tip culture in two *Impatiens* species. Physiological and Molecular Plant Pathology 79:79-88. doi: 10.1016/j.pmpp.2012.05.003

Ren HX, Wang ZL, Chen X, Zhu YL (1999) Antioxidative responses to different altitudes in Plantago major. Environmental and Experimental Botany 42:51-59. doi: 10.1016/s0098-8472(99)00015-5

Saez PL et al. (2019) Effects of temperature and water availability on light energy utilization in photosynthetic processes of Deschampsia antarctica. Physiologia Plantarum 165:511-523. doi: 10.1111/ppl.12739

Šmilauer P, Lepš J (2014) Multivariate Analysis of Ecological Data using CANOCO 5. Cambridge University Press., Cambridge.

Zhang FF et al. (2012a) Effects of CO_2_ enrichment on growth and development of *Impatiens hawkeri*. Scientific World Journal:1-9. doi: 10.1100/2012/601263

Zhang L et al. (2012b) Gene or environment? Species-specific control of stomatal density and length. Ecology and Evolution 2:1065-1070. doi: 10.1002/ece3.233
